# Early diagnostic markers of sepsis after oesophagectomy (including thromboelastography)

**DOI:** 10.1186/1471-2253-12-12

**Published:** 2012-06-28

**Authors:** Miroslav Durila, J Bronský, T Haruštiak, Alexander Pazdro, Marta Pechová, Karel Cvachovec

**Affiliations:** 1 Department of Anesthesiology and Critical Care Medicine, Second Faculty of Medicine and Institute for Postgraduate Medical Education, Charles University in Prague, V Úvalu 84, 150 06, Prague 5, Czech Republic; 2 Department of Paediatrics, Charles University, Second Faculty of Medicine, Prague, Czech Republic; 3 Third Department of Surgery, First Faculty of Medicine, Prague, Czech Republic; 4 Department of Clinical Biochemistry and Pathobiochemistry, Second Faculty of Medicine, Charles University, Prague, Czech Republic

**Keywords:** Sepsis, Biochemical, Hematological, Thromboelastography

## Abstract

**Background:**

Early diagnosis of sepsis and its differentiation from the noninfective SIRS is very important in order that treatment can be initiated in a timely and appropriate way. In this study we investigated standard haematological and biochemical parameters and thromboelastography (TEG) in patients who had undergone surgical resection of the oesophagus to find out if changes in any of these parameters could help in early differentiation between SIRS and sepsis development.

**Methods:**

We enrolled 43 patients (aged 41–74 years) of whom 38 were evaluable. Blood samples were obtained on the morning of surgery and then at 24-hour intervals for the next 6 days. Samples were analysed for procalcitonin (PCT), C-reactive protein (CRP), interleukin-6 (IL- 6), aspartate transaminase (AST), alanine transaminase (ALT) , lactate, white blood count (WBC), D-dimers, antithrombin (AT), international normalised ratio (INR), activated partial thromboplastin time (APTT) and parameters of TEG.

**Results:**

Significant differences between patients who developed sepsis during this period (9 patients) and SIRS were found in ALT on Day 1, in AST on Days 1–4, in PCT on Days 2–6; in CRP on Days 3–6; in IL-6 on Days 2–5; in leucocytes on Days 2, 3 and 6; and in D-dimers on Days 2 and 4. Significance values ranged from p < 0.0001 to p < 0.05.

**Conclusions:**

Sequential measurements of ALT, AST, PCT and IL-6 during the early postoperative period can be used for early differentiation of sepsis and postoperative SIRS after oesophagectomy. Among the coagulation parameters measured, only D-dimer concentrations appeared to be helpful in this process. TEG does not seem to be a useful early predictor of sepsis development; however it can be used to differentiate sepsis and SIRS from Day 5 after surgery.

## Background

Sepsis is a common, life-threatening condition in critically ill patients and, despite the availability of new therapeutic options for treating it, mortality rates remain high [1]. One reason for this may be delays in reaching a diagnosis and beginning treatment.

Patients undergoing major surgery often develop postoperative systemic inflammatory response syndrome (SIRS) in response to trauma, ischaemia, inflammation and/or infection. When due to an infection, SIRS may be self-limiting or may progress to severe sepsis [2]. In SIRS, proinflammatory cytokines induce intravascular coagulation and fibrinolysis is inhibited by production of plasminogen activator inhibitor 1 [3]. In septic patients this leads to hypercoagulability and the consumption of coagulation inhibitors and microthrombi formation with development of multiple organ dysfunction syndrome (MODS [4,5]. A marker that could distinguish an inflammatory septic response from inflammatory non-infective events would be helpful, therefore, to ensure that patients receive early treatment.

Biochemical markers that are believed to help in early diagnosis of sepsis include procalcitonin (PCT), interleukin 1 (IL 1), IL 6, IL 10 and C-reactive protein (CRP), although reports of sensitivity and specificity differ [6,7]. Thromboelastography (TEG) is a reliable method for evaluating hypercoagulability [8]. Unlike standard coagulation tests, such as prothrombin time (PT) or activated thromboplastin time (aPTT), TEG provides information about all phases of the coagulation process, from initiation of blood clot formation through fibrinolysis. Moreover, because whole blood is analysed, TEG takes account of interactions between all blood components (platelets, coagulation factors, leucocytes etc.) in the coagulation process.

Some authors have also identified markers of liver dysfunction in patients with SIRS or sepsis [9,10]. The mechanisms by which this occurs in sepsis involve the leaking of bacterial products into the systemic circulation, thus promoting the production of proinflammatory cytokines. Liver dysfunction can therefore be considered a part of SIRS which characterizes sepsis [11-13].

The aim of our study was to find early diagnostic marker of sepsis that might help to differentiate septic patients from patients with non-infective postoperative SIRS. We investigated changes in biochemical and hematological parameters during the early postoperative period, in patients who had undergone surgical oesophagectomy – a double cavity surgery, which has an accompanying high risk of postoperative complications, including sepsis, and is associated with high mortality rates [14].

## Methods

Forty three patients undergoing surgical oesophagectomy with a thoracoabdominal approach were included in this study. All patients received preoperative chemotherapy (epirubicin, cisplatin, 5-fluorouracil) ending 3 weeks before undergoing the operation. Exclusion criteria included hepatic insufficiency and coagulation disorders. This study was approved by the Ethics Committee for Multi-Centric Clinical Trials of the University Hospital Motol, Prague, Czech Republic. All patients provided informed consent before inclusion in the study.

Following surgery, all patients were extubated in the operating theatre and then transferred to the surgical intensive care unit (ICU). Post-surgical medications for all patients included antibiotics (for the first 48 hours), thoracic epidural analgesia, Diclofenac 1 x 75 mg/24 h and 1 x 0.4 ml (40 mg) Clexane (Enoxaparin) at 10 p. m.

Blood samples were obtained from the cubital vein using the syringe-needle method on the morning and of surgery and then at 24-hour intervals for the following 6 days. Samples were analysed for: PCT (Elecsys BRAHMS PCT; Roche Diagnostics, Mannheim, Germany); IL- 6 (EIA IL-6; IMMUNOTECH, Marseille, France); lactate (Cobas Integra 400+; Roche, Switzerland); CRP, aspartate transaminase (AST) and alanine transaminase (ALT) (all Advia 1800; Siemens, USA). White blood count (WBC) (Coulter LH 750 Hematology Analyzer; Beckman-Coulter, Miami, FL); and antithrombin (AT) activity, D-dimer concentration, international normalized ratio (INR) and activated partial thromboplastin time (aPTT) (all CA-7000 automated coagulation analyzer; Sysmex; Kobe, Japan). TEG analysis was conducted with a computer-controlled thrombelastograph haemostasis system (TEG Haemoscope, Niles, IL) using native cuvettes (without heparinase) and the native method (non-activated, without kaolin). Recently TEG is being validated as a tool for assessment of coagulation abnormalities in sepsis [15,16]

Patients who fulfilled the criteria of sepsis defined by ACCP-SCCM conference [17] during this period formed the ‘septic’ group. Other patients formed the ‘non-septic’ group. The ICU doctors, who were in charge of the definition and division of the patients into the groups, were blinded to the results of the study tests.

**(6)** The laboratory results of all investigated parameters mentioned above were compared between the two groups every day. For those parameters, in which singnificant diferences were found during the first 3 days, cutoff points for differentiations between sepsis and non-sepsis were set up.

### Statistical analysis

Statistical analysis was performed using Prism 5.0 statistical software (Graph Pad Software, Inc.). Values were tested for normality of distribution. Results are reported as mean ± SEM. We used parametrical tests for normally distributed data, unpaired t-tests for comparisons of the two groups, repeated-measures analysis of variance (ANOVA) with Bonferroni´s multiple comparison test for analyses within a group and the Pearson coefficient for correlation analyses. In non-normally distributed data, the differences between two groups were tested using the Mann–Whitney test and the Friedman test with Dunn´s multiple comparison for comparison of repeated measurements within a group. Correlations were expressed using the Spearman correlation coefficient. A P value of less than 0.05 was considered statistically significant. A receiver operating characteristics (ROC) curve was used for every parameter to find optimal value with best specificity and sensitivity with respect to discrimination of sepsis from non-sepsis.

## Results

In all, 43 patients were enrolled in the study: four patients were excluded because the surgery did not proceed as planned, and one patient withdrew from study. Among the evaluable patients, there were seven females and 31 males ranging in age from 41 to 74 years. Prior to surgery, the overall mean Acute Physiology and Chronic Health Evaluation II (APACHE II) score was 4 (range 0–9)., APACHE II scores tended to be higher in patients who went on to develop sepsis, but the scores were not significantly different to those in the non-sepsis group (mean 4.4 vs. 3.8, respectively; p = 0.36).

### Development of sepsis

All patients developed SIRS within 48 hours following surgery. Subsequently nine out of 38 patients (23.7 %) started to develop sepsis within the next 4 days after surgery. Five of these had pneumonia diagnosed from clinical symptoms (cough, expectorating coloured sputum and sepsis criteria) and confirmed by X-ray. In the four remaining cases dehiscence of the gastroesophageal anastomosis (diagnosed by radiology examination of the esophagus and confirmed by surgical revision of thorax) was the cause of sepsis. The median time to sepsis diagnosis was 3 days. According to our findings detectable changes in laboratory markers could be found as soon as 24–48 hours before diagnosis.

### Estimates of liver function

**Figure 1:**
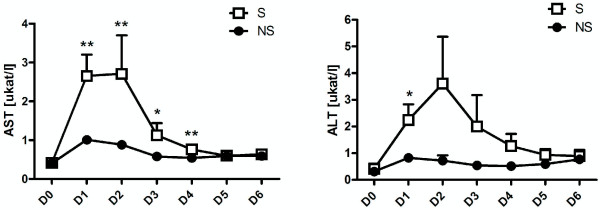
**Mean postoperative changes in serum AST (A) and ALT (B).** Changes from day of surgery (D0) to Day 6 after surgery (D6) in the septic (S) and nonseptic (NS) groups. There are significant differences between the S and NS groups from as early as the day after surgery. * indicates p < 0.05. ** indicates p < 0.01. ***indicates p < 0.001.

Changes in the levels of serum AST and ALT are shown in Figure 1A and 1B for 6-days period. Levels of AST and ALT in patients in the septic group were statistically significantly higher than in the non-septic groups from the 1-st day after surgery. For AST this difference persisted to Day 4 (values of p ranged from <0.002 to 0.006).

Cutoff points for early diagnosis of development of postoperative infectious complications within 6 days of surgery are shown in Tables 1, 2 and 3 (for the 1-st three days).

**Table 1:** Cutoff points (generated from ROC curves) for early diagnosis of postoperative infectious complication and its prediction within 6 days for Day 1 postsurgery

**Day 1**	**Cutoff**	**Sensitivity ( %)**	**Specificity ( %)**
AST ukat/l	>0.7	100	34.48
	>1.13	88.89	72.41
	>2.96	44.44	100
ALT ukat/l	>0.29	100	4.17
	>0.94	77.78	75
	>3.79	22.22	100

**Table 2:** Cutoff points (generated from ROC curves) for early diagnosis of postoperative infectious complication and its prediction within 6 days for Day 2 postsurgery

**Day 2**	**Cutoff**	**Sensitivity (%)**	**Specificity (%)**
AST ukat/l	>0.54	100	34.48
	>1.03	77.78	75.86
	>2.92	11.11	96.55
PCT ug/l	>0.25	100	3.45
	>3.18	77.78	75.86
	>5.65	55.56	96.55
IL-6 ng/l	>148.8	100	41.38
	>273.8	77.78	82.76
	>499.3	22.22	100

**Table 3:** Cutoff points (generated from ROC curves) for early diagnosis of postoperative infectious complication and its prediction within 6 days for Day 3 postsurgery

**Day 3**	**Cutoff**	**Sensitivity (%)**	**Specificity (%)**
AST ukat/l	>0.47	88.89	41.38
	>0.77	66.67	82.76
	>1.28	33.33	100
PCT ug/l	>0.28	100	13.79
	>2.02	77.78	79.31
	>3.31	55.56	96.55
IL-6 ng/l	>43.72	100	10.34
	>134.7	77.78	86.21
	>219.8	44.44	96.55
CRP mg/l	>143.3	100	48.28
	>174.6	66.67	68.97
	>269.5	11.11	100

### Biochemical parameters

**Figure 2:**
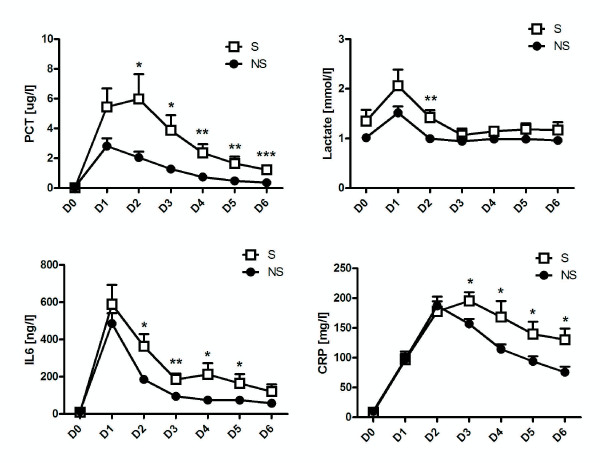
**Mean postoperative changes in serum PCT (A); lactate (B); IL-6 (C); CRP (D).** Changes from day of surgery (D0) to Day 6 after surgery (D6) in the septic (S) and nonseptic (NS) groups. PCT and IL-6 are statistically significant in the S and NS groups from D2. * indicates p < 0.05. ** indicates p < 0.01. ***indicates p < 0.001.

Figure 2 shows changes in serum levels of PCT, lactate, IL-6 and CRP. On Day 2 after surgery, levels of PCT (p = 0.03), IL-6 (p < 0.03) and lactate (p < 0.006) in patients in the septic group were statistically significantly higher than in the non-septic group. In the case of PCT, the magnitude of the difference increased over time, reaching p = 0.0001 at Day 6 (Figure 2A). IL-6 levels remained higher until Day 5 (Figure 2C) Differences in the levels of CRP in the septic and non-septic groups were statistically significantly different on Day 3 (p < 0.04) and remained so until Day 6 (Figure 2D).

Cutoff points for early diagnosis of development of postoperative infectious complications within 6 days of surgery are shown in Tables 2 and 3 (for days 2 and 3 after surgery).

### Haematological and coagulation parameters

**Figure 3:**
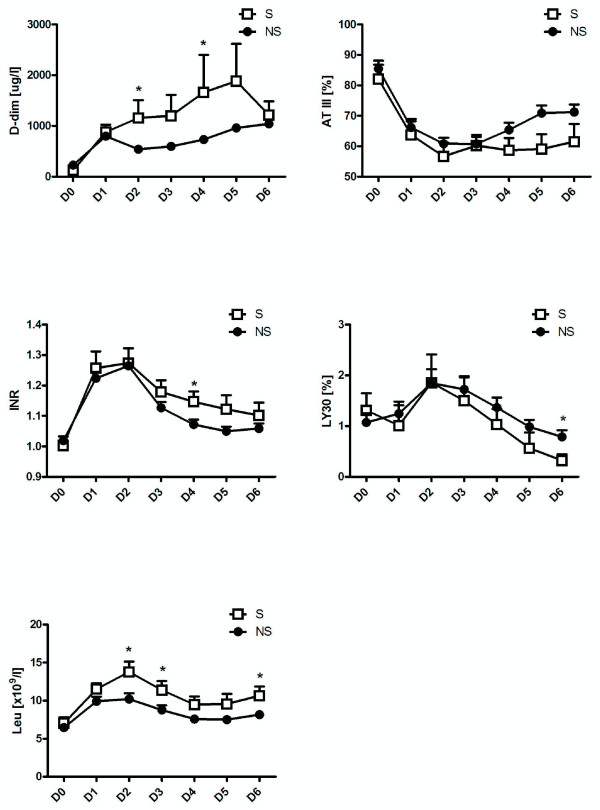
**Mean postoperative changes in serum D-dimers (A); ATIII (B); INR (C); LY-30 (D); and leucocytes (E).** Changes in concentrations of haematological and coagulation factors in the septic (S) and non-septic (NS) groups from day of surgery (D0) to Day 6 after surgery (D6). Suprisingly, TEG is not an early predictor of sepsis development. * indicates p < 0.05.

Changes in serum levels of LY30 (ie the percentage of coagulum lysed in the TEG analysis at 30 minutes after the maximal strength of clot), D-dimers AT III, INR and leucocytes are shown in Figure 3. Differences in serum concentrations of leucocytes in the septic and non-septic groups were just statistically significantly different on Days 2 (p < 0.03), 3 (p < 0.05) and 6 (p < 0.03) (Figure 2E). Statistically significantly higher concentrations of D-dimers were found on Days 2 and 4 (p < 0.02 and p = 0.03, respectively). Levels of INR, AT III and LY30 were statistically significantly higher in the septic group on Days 4, 5 and 6 respectively (p = 0.03 for INR and AT III; p < 0.04 for LY30).

## Discussion

Patients who have undergone major surgery are at high risk of developing complications associated with sepsis [14,18]; however, differentiation between postoperative SIRS and septic complications is difficult. Long-term prophylaxis with high-dose antibiotics or wide-spectrum antibiotics, which might prevent the development of sepsis, may obscure the host response and induce the development of resistance. Identification of septic patients at the beginning of the process can help in better and more specific diagnostic and therapeutic procedures and overall management. On the contrary, exclusion of sepsis might help in better and rationale antibiotics treatment.

In our study, we investigated both hematological (including TEG) and biochemical markers of sepsis, as there is a link between inflammation and coagulation (5). We also examined markers of liver dysfunction because of the important role reported to be played by the liver in sepsis. This is a very interesting finding and is, in all probability, underestimated in practice. Interestingly, levels of AST and ALT were significantly higher in the septic group of patients compared with the nonseptic group, and those changes could be detected as early as the first day after surgery and persisted to the fourth day (see Figure 1). For cutoff points for early diagnosis of development of postoperative infectious complications within 6 days of surgery see Tables 1, 2 and 3.

Our findings of higher liver enzymes levels in septic patients are in accordance with findings of others who say that liver dysfunction is common in patients with sepsis, ranging from mild elevations of serum aminotransferases to severe cholestasis [11-13,19,20]. However, we think that in our case liver dysfunction might rather be a cofactor participating in sepsis development. Liver has a central role in the regulation of host defenses. It serves as a source of inflammatory mediators and is a major site of the removal of bacteria and endotoxins from systemic circulation. Kupffer cells (KCs) of the liver make up 80 %-90 % of the fixed-tissue macrophages of the reticuloendothelial system. KCs take up bacteria, endotoxins and are stimulated to release a wide range of products implicated in liver injury, such as tumor necrosis factor, interleukin-1 and interleukin- 6 [12]. It is proposed that Kupffer cell phagocytic depression associated with liver dysfunction permits spread of endotoxin and inflamatory mediators and thus predisposes to sepsis and multiple organ failure.

The cause of postoperative liver dysfunction in our patients is not known. All patients had received the same kind of anaesthesia (isofluran, remifentanil, rocuronium) and postoperative care and there are some reports of isofluran side effect on liver function [21]. Transient hypotension and hypoxia cannot be ruled out and individual response of the patient to surgery can also play a part. That could have played a role in development of ,,postanaesthetic hepatitis” and subsequently in development of sepsis. Importantly, unlike the expensive tests used for detection of inflammatory markers, measurements of liver dysfunction are cheap and routinely available and can be helpful in screening of patients susceptible to sepsis for early signs of sepsis development.

Overall, the earliest markers of the development of sepsis seem to be AST and ALT. Differences in PCT and IL-6 in septic and non-septic patients with SIRS are detectable from Day 2 (see Figure 2). For cutoff points for early diagnosis of development of postoperative infectious complications within 6 days of surgery see Tables 2 and 3. Both parameters showed similar trends on subsequent days, which means that, as IL-6 is measured mostly experimentally, measurements of PCT should be sufficient to provide the necessary information. Ito and colleagues actually found that PCT is better for monitoring the development of sepsis in patients who had undergone oesophageal surgery for carcinoma; however they were able to predict sepsis one day earlier than in our study [22]. Unlike our patients though, their patients did not receive preoperative chemotherapy. In another study, Mokart and colleagues investigated PCT and IL-6 to predict sepsis development in patients who had undergone gastrointestinal or gynaecological tumour resections. They were also able to predict the development of sepsis from as early as Day 1 after surgery, although levels of IL-6 and PCT were not as high as those of our patients [7]. It should be noted, however, that unlike our patients, those patients had only undergone surgery in the abdominal cavity.

When it comes to CRP, we found significant differences between the groups from Day 3, when CRP levels in the septic group rose by comparison with those of patients in the non-septic group (see Figure 2). For cutoff points for early diagnosis of development of postoperative infectious complications within 6 days of surgery see Table 3. This finding is similar to that described by Ito, although the differences were not statistically significant in that study. Although it is questionable just how valuable this marker is for early diagnosis of sepsis, the tests are cheap and can help in sepsis diagnosis.

Surprisingly, among the coagulation parameters, only concentrations of D-dimers were statistically significantly higher during the early postoperative period (from Day 2 to Day 4). However, since the concentrations of D-dimers subsequently decreased (Figure 3A), the value of this marker for early diagnosis of sepsis is questionable.

Because this kind of operation is not very often, the sample size might be the limitation of this study. However, we were able to find some interesting laboratory changes in patients who would develop infectious complication untill the 6-th postoperative day, as soon as the 1-st postoperative day, no matter if sepsis was diagnosed on day 3 or 6. This findings could attract our attention to those patients in the risk of sepsis development more then 24 hours before sepsis diagnosis.

**Figure 4:**
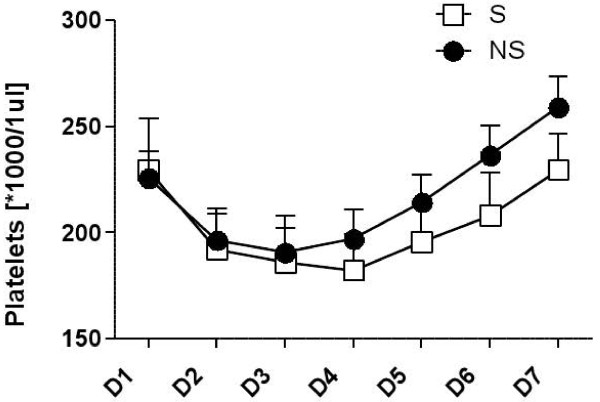
**The evolution and changes in platelet counts and fibrinogen level over time in the septic (S) and non-septic (NS) groups from day of surgery (D0) to Day 6 after surgery (D6).** No significant differences. *means sign of multiplication.

We also evaluated changes in pletelets count and fibrinogen level in both groups during the investigation time period, but there was no significant difference between their values (p > 0.05) (Figure 4).

Our finding of decreased fibrinolytic activity (represented by levels of LY30) in the septic group versus the non-septic group is in accordance with those of a recent study by Adamzik and colleagues. This group investigated coagulation changes in septic patients using TEG and also found decreased fibrinolytic activity [23]. In that study, TEG did not detect changes until 24 hours after severe sepsis had been diagnosed and the patients were in a much more serious condition than ours. In both our study and the Adamzik study then, patients had already developed sepsis before TEG detected changes in fibrinolysis. Thus, while TEG is useful to diagnose sepsis in later state, it is not useful for early diagnosis of sepsis or predicting which patients will go on to develop sepsis.

## Conclusions

Sequential measurements of ALT, AST, PCT and IL-6 during the early postoperative period can be used for early differentiation of sepsis and postoperative SIRS after oesophagectomy. Among the coagulation parameters measured, only D-dimer concentrations appeared to be helpful in this process. TEG does not seem to be a useful early predictor of sepsis development; however it can be used to differentiate sepsis and SIRS from Day 5 after surgery.

We can conclude that sequential measurements of biochemical parameters, such as AST, ALT, PCT and IL-6, during the early postoperative period can be useful for the early diagnosis of sepsis development and its prediction following oesophagectomy, and to differentiate sepsis from postoperative SIRS. Among coagulation parameters, only D-dimer concentrations appeared to be helpful in this process. By contrast, TEG does not seem to be helpful as an early diagnostic marker of sepsis development; however, it can be used to differentiate between sepsis and SIRS from Day 5 after surgery.

## Competing interests

All authors declare that they have no competing financial or other interest in relation to their work.

## Authors’ contributions

All authors have made substantial contributions to conception and design of the study, participated in acquisition, analysis and interpretation of data. All of them have been involved in drafting the manuscript and revising it critically for important intellectual content; and have given final approval of the version to be published. All authors read and approved the final manuscript.

## Pre-publication history

The pre-publication history for this paper can be accessed here:

http://www.biomedcentral.com/1471-2253/12/12/prepub
